# Thiamine pyrophosphate may protect indomethacin-induced small intestinal enteropathy in rats by inhibiting intestinal inflammation and oxidative stress

**DOI:** 10.1007/s00210-025-04213-9

**Published:** 2025-04-29

**Authors:** Seyda Secgin, Mudassar Zafar, Mukhammadiy Khakimov, Hikmet Keles, Tahir Kahraman

**Affiliations:** 1 Department of Biochemistry, Faculty of Medicine, Karabuk University, 78050 Karabuk, Turkey; 2 Department of Pathology, Faculty of Veterinary Medicine, Afyon Kocatepe University, 03030 Afyonkarahisar, Turkey

**Keywords:** Nonsteroidal anti-inflammatory drugs, Indomethacin, Small intestine, Thiamine pyrophosphate, Inflammation, Oxidative stress injury

## Abstract

Non-steroid anti-inflammatory drug (NSAID)-induced enteropathy is a clinically undesirable but highly prevalent problem. Thiamine pyrophosphate (TPP) has a role in reducing oxidative stress as well as acting as a coenzyme in enzymatic reactions. The aim of this study was to investigate the protective effect of TPP on the damage caused by indomethacin (IND), one of the NSAID group drugs, in the small intestine. In the experiment in which 32 rats were used, four groups were formed randomly (groups 1, 2, 3, 4; control, TPP, IND, IND + TPP group, respectively). Small intestinal injury was induced in group 3 and group 4 rats that were fasted 1 day beforehand by a single intragastric administration of 25 mg/kg IND. Half an hour before the model was created, 20 mg/kg TPP was administered to group 2 and group 4 by intragastric as pretreatment. Tissue changes, proinflammatory cytokines, oxidative stress status, macroscopic appearance, and histopathologic analysis were evaluated. All data were statistically analyzed, and significance was determined (*p* < 0.05). Prophylactic treatment with TPP resulted in maintenance of antioxidant enzymes (GPX, SOD) and GSH levels in small intestinal tissue analysis. However, the excessive increase in IND-induced lipid peroxidation (MDA) and total oxidant level (TOS) was not downregulated by TPP compared to group 3. Additionally, the treatment had no prophylactic effect on the reduction of proinflammatory cytokine (TNF-α, IL-6) levels in tissue. Histopathologic examination of the tissue revealed that IND disrupted the intestinal villus structure, causing erosive ulceration, degeneration, and inflammation. TPP reduced the inflamed areas and total tissue damage score in the IND group (*p* < 0.001). In this study, the positive effects of TPP use on some parameters in a short period of time suggested that TPP may produce more significant results with changes in the time and dose of use. Indeed, these beneficial effects obtained with a single dose suggest that TPP may provide protection in small intestinal enteropathy as an antioxidant and anti-inflammatory agent.

## Introduction

Non-steroid anti-inflammatory drugs (NSAIDs), which are on popular drug lists around the world, are generally used for analgesic, anti-inflammatory, and antipyretic purposes in chronic pain or postoperative pain and autoimmune diseases. Due to the high rate of use, the associated adverse effects are very common (Mahdavi et al. 2020; Cervantes-García et al. 2020). NSAIDs metabolically inhibit the synthesis of prostaglandins (PGE) from arachidonic acid in the inflammatory state. They are classified into different categories according to those that cause cyclooxygenase- 1 (COX- 1) and cyclooxygenase- 2 (COX- 2) inhibition or plasma half-life. Indole acetic acid derivative indomethacin (IND) acts in a similar mechanism to other NSAIDs, with both therapeutic effects and adverse effects mediated by reversible COX inhibition (Lucas 2016; Hahm and Shin 2019; Kraiem et al. 2024). Peptic ulceration, dyspepsia, perforation, and bleeding are the most common adverse effects in the gastrointestinal tract (Cervantes-García et al. 2020). NSAID-associated enteropathy is more common than gastric and duodenal complications and usually presents with occult blood loss or hyperuremia (Tai and McAlindon 2021). Much research has been and continues to be conducted to treat and prevent all these complications.

The side effects of NSAIDs primarily involve topical effects, including drug interaction with membrane phospholipids during absorption and inhibition of mitochondrial oxidative phosphorylation and inhibition of oxidative phosphorylation by causing tight junction protein (TJP) dysfunction. These topical effects increase intestinal permeability (Cervantes-García et al. 2020; Han et al. 2020). As it is known, PGE is a mediator that plays an important role in the repair of the mucosa. As a matter of fact, the damage to the mucosa caused by the decrease in PGE production caused by IND increases intestinal permeability (Tai and McAlindon 2018b). IND in particular is the drug that increases intestinal permeability the most of all NSAIDs. Increased permeability can cause bacterial toxins and lipopolysaccharides to pass into the bloodstream, leading to systemic inflammation, as well as local inflammation in enterocytes (Tai and McAlindon 2018b; Han et al. 2020). In erosive ulcerations and inflammation caused by mucosal damage, luminal contents (bile-pancreatic secretions, dietary macromolecules, bacteria in the intestinal microbiome (especially gram-negatives)) act by increasing neutrophil chemotaxis (Tai and McAlindon 2018b; Cervantes-García et al. 2020; Han et al. 2020). It has also been reported that another element in the pathophysiology of IND enteropathy is the increase of excessive reactive oxygen species (Kunikata et al. 2002; Turkyilmaz et al. 2019; Küçükler et al. 2022).

Thiamine pyrophosphate (TPP), which we used in IND enteropathy in our study, is the active form of vitamin B1 (thiamine) in the liver. TPP acts as a coenzyme in the production of nicotinamide adenine dinucleotide phosphate (NADPH) in the pentose phosphate pathway (Turan et al. 2013b). Therefore, the intracellular antioxidant substance plays an important role in maintaining the intracellular antioxidant balance indirectly by providing NADPH production, which is the precursor in the synthesis of glutathione (Altuner et al. 2013; Turan et al. 2013b). In a study comparing the antioxidant effect of TPP and thiamine, TPP had an effect on malondialdehyde (MDA), myeloperoxidase (MPO), glutathione (GSH), glutathione peroxidase (GPX), glutathione transferase, and superoxide dismutase (SOD), while thiamine had no effect (Turan et al. 2013a). In the studies conducted by Halis et al., the antioxidant effect of TPP was demonstrated with many biomarkers in different oxidative stress conditions in experimental animals (Demiryilmaz et al. 2012; Yapca et al. 2014; Acun Delen et al. 2022). Based on the idea that TPP has this property, the aim of this study was to explain the protective effect of TPP in IND-induced small intestinal complications biochemically and histopathologically.

## Material method

### Ethical approval and experimental animal design

The approval of the Karabuk University Experimental Animals Local Ethics Committee was obtained for the experimental applications of the study. In addition, experimental animal applications were to be performed at the Karabuk University Experimental Application and Research Center, biochemical analyses were to be performed at the Karabuk University Medical Biochemistry laboratory, and histopathological analyses were to be performed at Afyon Kocatepe University Veterinary Pathology laboratory. In the study, 32 Wistar females, approximately 15 weeks old and weighing 160–200 g, which had completed their adaptation to the environmental conditions, were included. Rats were randomly divided into four groups (*n* = 8).

### Establishment of intestinal animal model and pretreatment with indomethacin

**Fig. 1:**
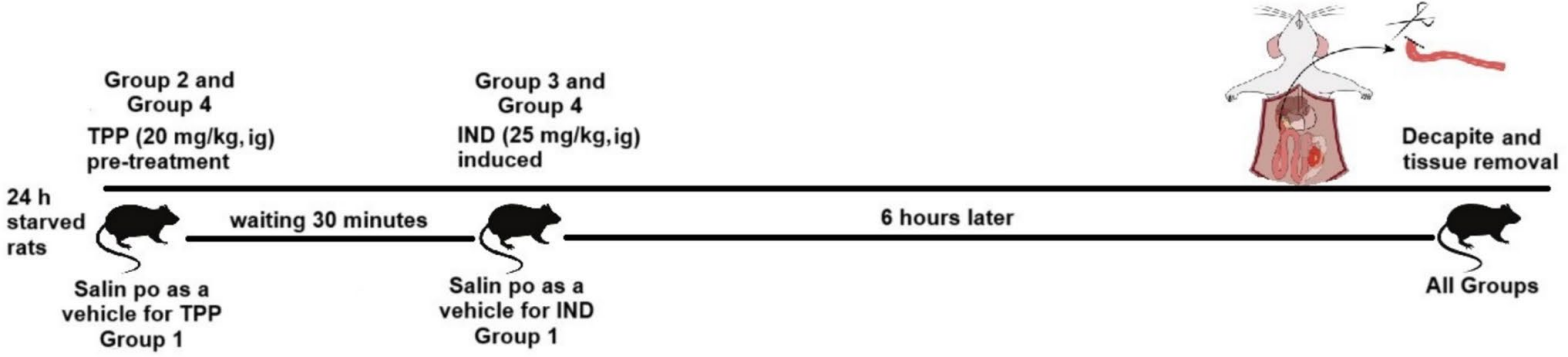
Timeline of the treatment of experimental animals. Rats were divided into four groups (*n* = 8), and group 1 was used as control (vehicle was used as control). The second group received TPP (20 mg/kg intragastric) on the day of the experiment. In the third group, intestinal injury was induced with IND (25 mg/kg, intragastric). In the fourth group, TPP (20 mg/kg intragastric), whose prophylactic effect was examined, was administered first. After 30 min, IND 25 mg/kg intragastric, which causes intestinal damage, was administered. The animals were euthanized 6 h after IND administration, and small intestinal tissues were removed. TPP, thiamine pyrophosphate; IND, indomethacin

After adaptation to the environment was completed, the rats were deprived of feed the night before to keep their gastrointestinal contents empty and were given free access to water. On the day of the experiment, 20 mg/kg TPP was administered intragastrically to group 2 and group 4 for pretreatment (Yapca et al. 2014). Half an hour later, indomethacin 25 mg/kg was administered intragastrically to groups 3 and 4. It is reported in the literature that 25 mg/kg indomethacin was used as an experimental animal model by causing inflammation in the intestines (Turkyilmaz et al. 2019). It is also reported in the literature that this dose causes complications in the gastrointestinal system such as excessive gastric ulcer and bleeding in the stomach, which precedes the small intestine (Boyacioglu et al. 2016). In the control group, IND and TPP solvents, 0.9% NaCl were administered intragastrically at appropriate times (Fig. 1).

Six hours after IND administration, animals were anesthetized with xylazine/ketamine (80/50 mg/kg, Fig. 1). Immediately afterwards, small intestinal tissues were removed, and animals were euthanized by removing blood from the heart. The intestinal contents were then cleaned two to three times with cold phosphate buffer solution (PBS, pH:7.4) buffer without damaging the lumen. For each animal, a portion of the small intestine was stored in the deep freezer until the day of analysis for biochemical analyses. The remaining parts of the small intestine were soaked with formaldehyde on a flat surface to prevent curling and placed in 10% formaldehyde for histopathological analysis after hardening.

### Biochemical analysis

Samples stored in a deep freezer for biochemical analyses were gradually reduced in temperature on the day of the experiment, and approximately ten times the weight of the wet tissue was added in cold PBS (Ph 7.4). Homogenization was performed at 12,000 rpm for 2 min (IKA, Germany). The homogenates obtained were used for MDA, GSH, SOD, GPX, total antioxidant level (TAS), total oxidant level (TOS), tumor necrosis factor-α (TNF-α), and interleukin- 6 (IL- 6) analysis. MDA level analysis was performed from the homogenate, and other parameters were analyzed from the supernatant after centrifugation of the homogenate at 4000 rpm for 15 min.

Beutler method was used for GSH determination in small intestinal tissues (Beutler and Kelly 1963). In this method, all proteins without -SH group were precipitated and the yellow color formed by 5.5′-dithi- 2-nitrobenzoic acid (DTNB) was read colorimetrically at 412 nm wavelength. MDA, the most stable lipid peroxidation product, was determined by reading the absorbance of the pink color formed by the reaction of MDA with thiobarbituric acid reagent at a wavelength of 532 nm (Ohkawa et al. 1979).

Small intestinal rat tissues were processed for SOD (Cat. no. E0168Ra), GPX (Cat. no. E1242a), TNF-α (Cat. no. E0764Ra), and IL- 6 (Cat. no. E0135Ra) ELISA kit (BT Lab, Zhejiang, China), according to the manufacturer’s protocol. Absorbance was read at 450 nm on a 96-well plate microplate reader (Thermo Scientific Multiskan Go, Finland).

Total antioxidant level (TAS Rel Assay Diagnostics, Lot no: TZ23142 A, Gaziantep, Turkey) and total oxidant level (TOS Rel Assay Diagnostics, Lot no: TZ23156O, Gaziantep, Turkey) in small intestinal tissue were determined by commercial rat kits. In the TAS kit, the antioxidant level was determined by reacting the blue-green 2,2″-azinobis- 3-ethylbenzothiazoline- 6-sulfonic acid (ABTS) reagent with thiol groups in the sample and reading the color change in the reaction at 660 nm. The results were calibrated to Trolox, a vitamin E analog, and calculated in mmol Trolox Edv/L. In the TOS kit, the oxidants in the sample were measured spectrophotometrically at 560 nm at 10-min intervals by the color change reaction resulting from the oxidation of Fe + 2 ion to Fe + 3 ion. The results were calibrated with hydrogen peroxide (H2O2) and expressed as μmol H2O2 Equiv/L. TAS and TOS spectrophotometric measurements were performed using the protocol recommended by the manufacturer and a microplate reader (Thermo Scientific Multiskan Go, Finland).

Total protein levels were determined in 1 ml/mg tissue by the Bradford method (Kruger 1994). Briefly, in this method, the positive charge on the proteins is bound to the minus charged Coomassie Brilliant Blue G- 250 used in the method, and the blue color resulting from the binding is read at a 595 nm wavelength. Bovine serum albumin is used as the standard. The results of other parameters obtained as a result of biochemical analysis are calculated per protein by using the protein levels obtained in this method.

### Microscopic examination of damage to the small intestine

The ileum tissue samples of rats were used for histopathological analysis. The formalin-fixed small intestine tissues were then embedded in paraffin and blocked. Sections taken from paraffin blocks were analyzed using Hematoxylin and Eosin staining using a magnification bar of 100 µm (micron), × 10 magnification. Ulceration, villus degeneration/necrosis, and inflammation were scored as normal tissue (−/0), mild lesions (+/1), moderate lesions (+ +/2), and severe lesions (+ + +/3) in the light microscopic examination of the tissues.

### Statistical analysis

SPSS 27.0 and Minitab 21 software programs were used in the analyses. One-way ANOVA (post hoc; multiple comparison tests using Tukey and Dunnett T3, results mean ± standard deviation) analysis of variance was performed for normally distributed data and Kruskal–Wallis H test (post hoc; pairwise comparison multiple comparison tests, results median (min–max)) for non-normally distributed data according to the results of the Shapiro–Wilk normality test. Statistical significance between groups was accepted as *p* < 0.05.

## Results

### Antioxidant evaluation of TPP in small intestinal injury

**Fig. 2:**
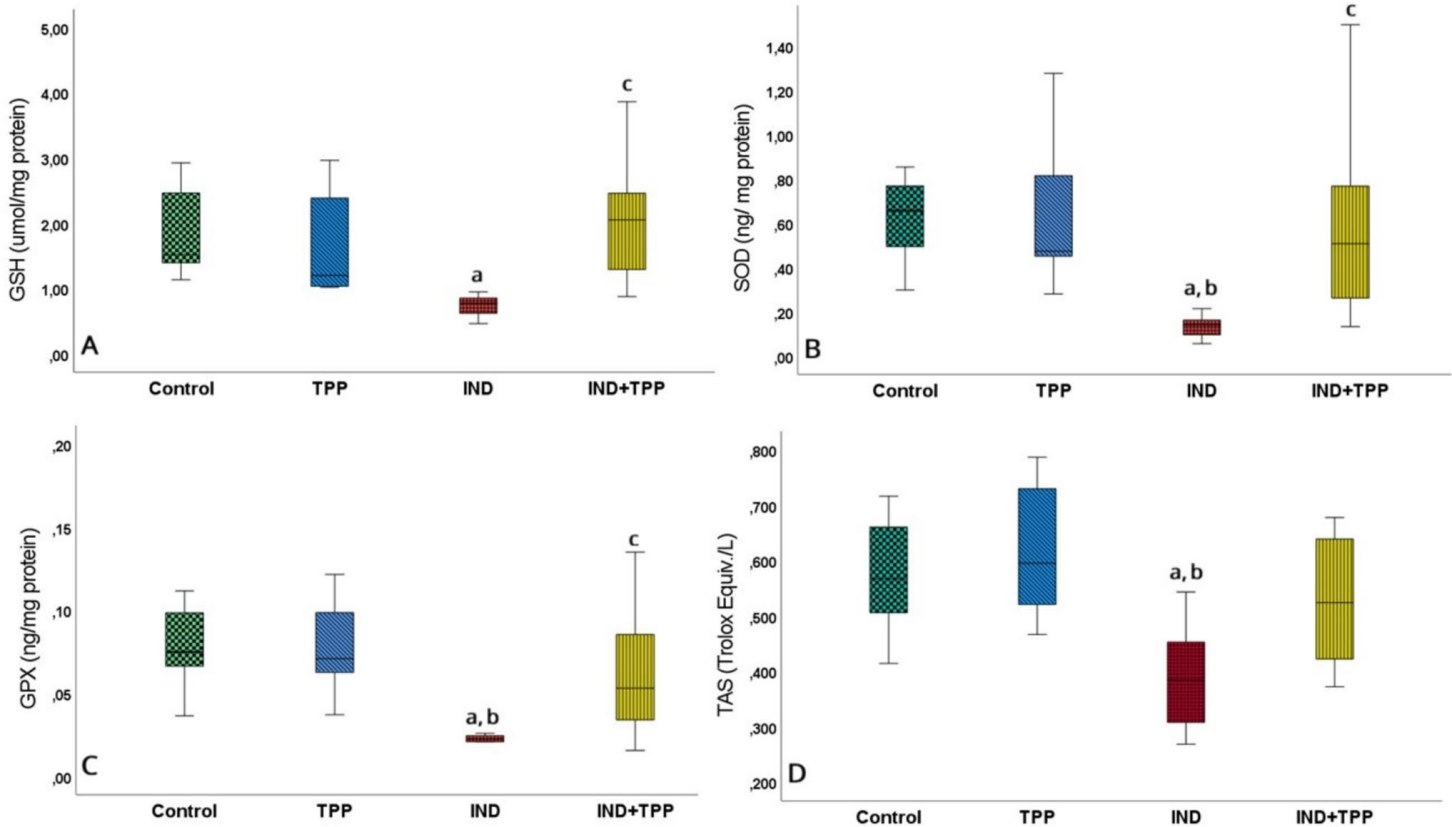
Effect of TPP on antioxidant balance in small intestine injury. **A, B, C, D**, respectively: intracellular tripeptide glutathione (GSH) antioxidant, antioxidant enzymes superoxide dismutase (SOD), glutathione peroxidase (GPX), and tissue total antioxidant levels (TAS). GSH, SOD, GPX, and TAS increased with indomethacin (IND, *p* < 0.05). Thiamine pyrophosphate (TPP), pretreatment did not decrease the antioxidant balance in the IND group. Values are given as mean ± standard deviation. a, b, c, d indicate significance compared to control, TPP, IND, and IND + TPP groups, respectively (*p* < 0.05, *n* = 8)

NSAID group drugs damage the epithelial cells of the small intestine. Intracellular GSH was found to be lower in rats given IND 25 mg/kg compared to the control group (Fig. 2A, *p* < 0.05). Antioxidant enzymes GPX and SOD were significantly decreased in the IND group compared to the control group (Fig. 2B, C; *p* < 0.05). In the IND + TPP group in which TPP was administered upfront, the amount of these enzymes was higher compared to the IND alone group (Fig. 2B, C; *p* < 0.05). The lowest tissue TAS level was measured in the IND group and was very low compared to the control (Fig. 2D, *p* < 0.05), but there was no significant increase in TAS level of TPP compared to IND + TPP group (Fig. 2D, *p* > 0.05).

### Evaluation of the effect of TPP on lipid peroxidation and oxidative stress in the small intestine

**Fig. 3:**
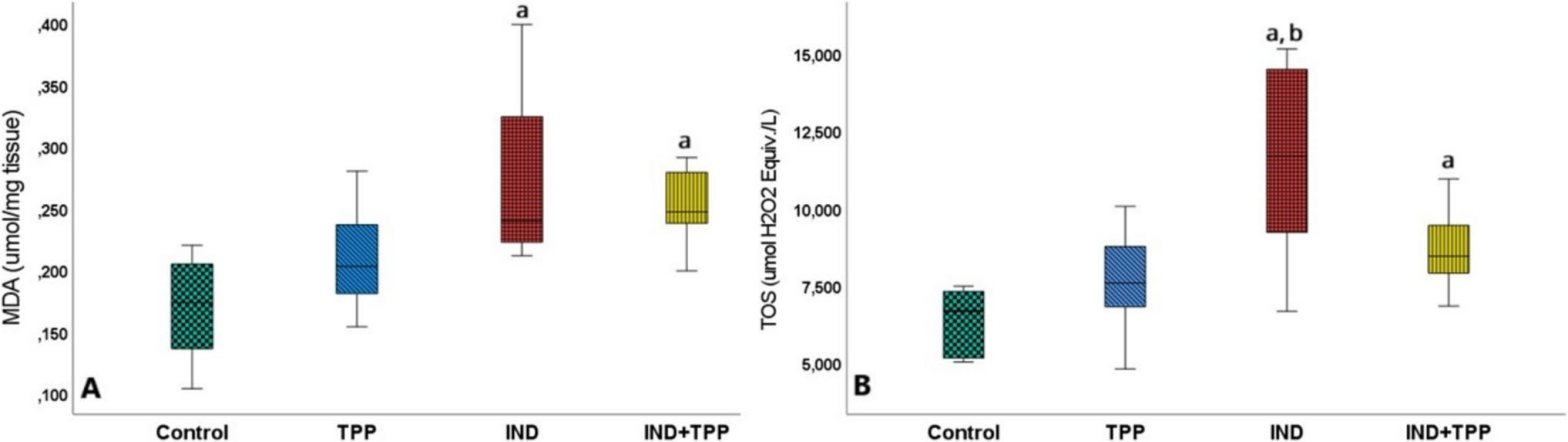
Oxidation and peroxidation status of TPP in small intestine injury. **A** The lipid peroxidation product malondialdehyde (MDA) and **B** tissue total oxidant capacity (TOS). MDA and TOS were considerably increased (*p* < 0.05) by indomethacin (IND) and not decreased by thiamine pyrophosphate (TPP, *p* > 0.05). Values are given as mean ± standard deviation. a, b, c, and d denote significance according to control, TPP, IND, and IND + TPP groups, respectively (*p* < 0.05, *n* = 8)

MDA and TOS were significantly higher in the small intestinal tissue after IND exposure compared to the control group (Fig. 3A, B; *p* < 0.05). However, there was no change in MDA level in the group pretreated with TPP (IND + TPP) compared to the IND group (Fig. 3A, *p* > 0.05). The same situation was also observed at the TOS level (Fig. 3B, *p* > 0.05).

### Evaluation of the effect of TPP on IND-induced damage in the small intestine with proinflammatory markers

**Fig. 4:**
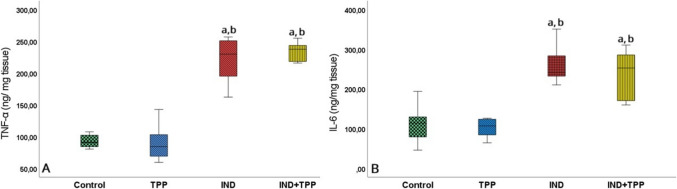
Effect of TPP on inflammation in small intestinal injury. **A** Tumor necrosis factor (TNF-α) and **B** inflammatory cytokine- 6 (IL- 6) proinflammatory mediator levels. Indomethacin (IND) increased TNF-α and IL- 6 levels in the small intestine, whereas thiamine pyrophosphate (TPP) pretreatment had no effect. Values are given as mean ± standard deviation. a, b, c, and d denote significance according to control, TPP, IND, and IND + TPP groups, respectively (*p* < 0.05, *n* = 8)

TNF-α and IL- 6 levels measured in small intestinal tissue were significantly higher in both IND-treated groups compared to the control group (Fig. 4A, B; *p* < 0.05). No difference was found between the TPP and control group in either biomarker. In parallel with this result, no significant difference was observed in proinflammation markers in the IND + TPP group compared to the IND group (Fig. 4A, B; *p* > 0.05).

### Histopathologic evaluation of TPP in the small intestine

**Fig. 5:**
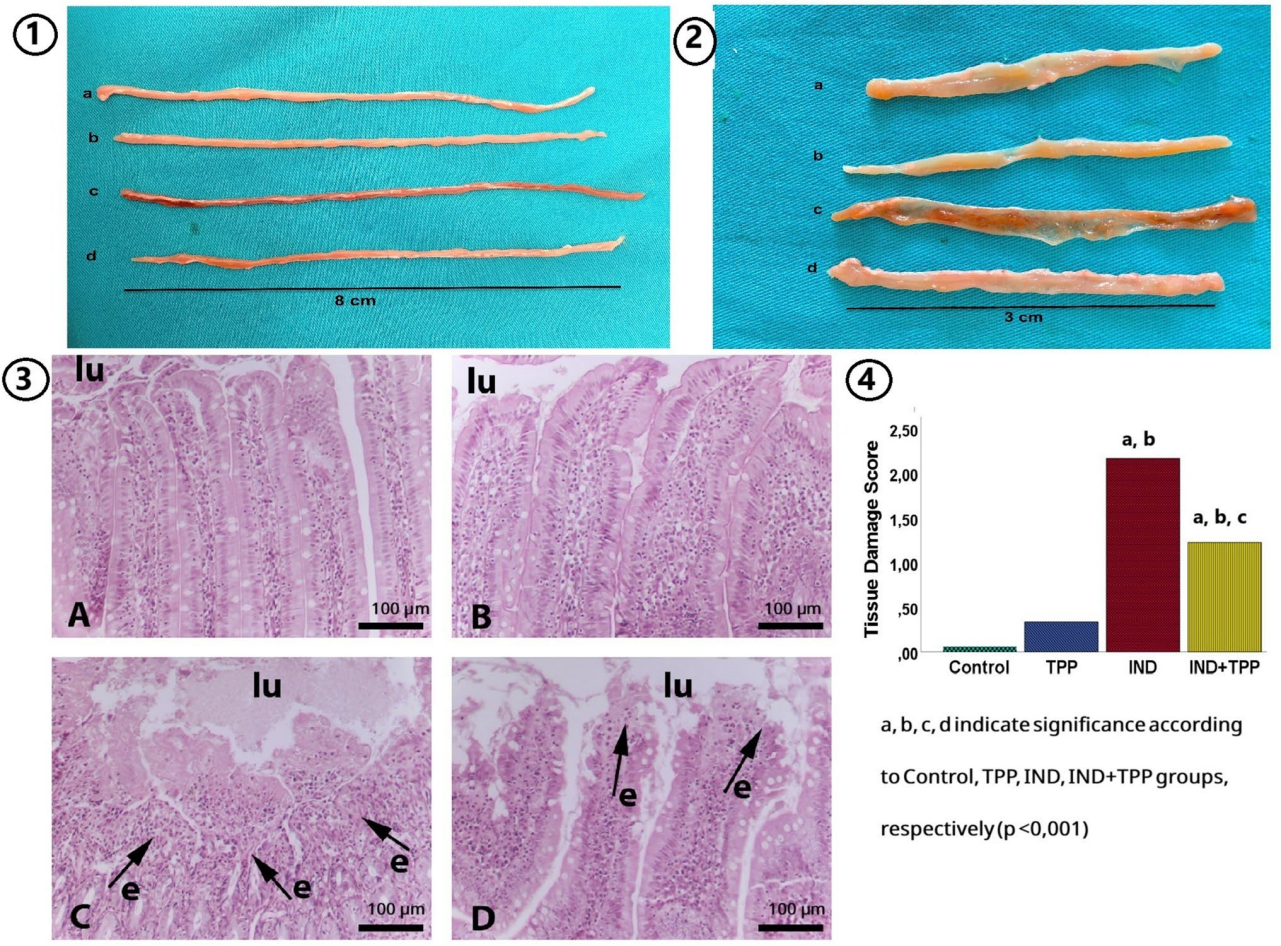
**1** Macroscopic image of the small intestine in an 8 cm section, free of luminal contents (a: control, b: TPP, c: IND, d: IND + TPP).** 2** View of the small intestine evaluated by opening the lumen. **3** Intestinal histopathology in control group and animals treated with TPP and IND, Hematoxylin and Eosin staining, × 10 magnification, magnification bar 100 µm (micron). A: Control group, normal intestine. B: TPP group, normal intestine. C: IND group, erosive and inflammatory findings in the intestine. D: TPP + IND group, erosive findings in the intestine, milder than the IND group. Findings shown with arrows; lu: intestinal lumen/the apical surface where the intestinal villi are located; e: erosion/superficial loss of epithelial tissue.** 4** Total damage score of pathologic findings in groups belonging to histopathologic examination. TPP, thiamine pyrophosphate; IND, indomethacin

Histologic sections were evaluated using standard light microscopy. In the histologic examination of the small intestine, the intestinal tissue in the control and TPP groups was significantly better than in the IND and IND + TPP groups. The presence/severity of damage in the small intestines of the rats was evaluated as degeneration/necrosis (disruption of villus architecture, decrease in villus length-crypt depth), ulceration, inflammation, degeneration/necrosis, and inflammatory findings (Fig. 5, Table 1). In the damage scoring measured by the pathologist, the IND group scored the highest with a significant difference compared to the other groups (Fig. 5 (4), *p* < 0.001). The IND + TPP group also had milder findings of ulceration, inflammation, and villus degeneration/necrosis (Fig. 5 (3D)). There was a significant difference in the total damage score of all findings between the IND and IND + TPP groups (*p* < 0.001).

**Table 1:** Histopathologic examination of the small intestine

Groups	Ulcerative erosion	Inflammation	Degeneration/necrosis	Total damage score
Control	0 ± 0	0.17 ± 0.16	0 ± 0	0.06 ± 0.1
TPP	0.33 ± 0.21	0.17 ± 0.16	0.5 ± 0.22	0.36 ± 0.14
IND	2.33 ± 0.33 ^a,b^	2.50 ± 0.22 ^a,b^	1.67 ± 0.21^a,b^	1.48 ± 1.21^a,b^
IND + TPP	1.33 ± 0.21^a, b^	1 ± 0.1^a,b,c^	1.33 ± 0.21^a^	1.32 ± 0.2^a,b,c^

Pathologic findings seen in light microscopy examination were scored as normal tissue (−/0), mild lesions (+/1), moderate lesions (+ +/2), and severe lesions (+ + +/3). a, b, c, and d denote significance according to control, TPP, IND, and IND + TPP groups, respectively (*p* < 0.001)

.

## Discussion

NSAIDs cause mucosal lesions in the intestines as well as the most common gastric ulcerations in humans. Glucocorticoids, aminosalicylates, and glucocorticoids are used to relieve inflammation in the healing of these lesions in the clinic (Bindu et al. 2020). Apart from medical drugs, researchers are working on alternative products with antioxidant and anti-inflammatory properties. The function of alternative products is evaluated according to the level of lipid peroxidation, oxidative stress, and inflammation that cause mucosal ulcerations (Bindu et al. 2020; Sánchez-Trigueros et al. 2024). In this study, for pretreatment of acute IND-induced small intestinal injury, TPP, the active form of vitamin B1, which is reported to have an antioxidant effect against oxidative stress, was administered to demonstrate its possible positive protective effect.

It is important to understand the etiology of NSAID-induced small intestine injury and to demonstrate prophylactic and therapeutic conditions for the disease. However, there is no known practice other than the use of proton pump inhibitors (PPIs). The use of PPIs has been reported in recent studies to have variable protective effects in individuals for unclear reasons (Han et al. 2020). Focusing on the gastroprotective effect of PPIs, NSAIDs are often prescribed together (Singh et al. 2016). Although PPIs are reported to reduce gastroduodenal damage, there is also the fact that the severity of damage in the small intestine increases. This fact has been reported in both clinical and experimental studies (Maiden et al. 2005; Singh et al. 2016; Tanigawa et al. 2021). PPIs have been reported to be a contributing cause of NSAID mucosal damage, with a synergistic effect, as PPIs cause reduced microbiome diversity in small intestinal tissue (Tanigawa et al. 2021). One of the important gaps in the literature is that although NSAID-induced enteropathy is thought to be caused by altered intestinal permeability, dysbiosis, and oxidative stress (Han et al. 2020), there are not enough studies aiming to elucidate the underlying mechanism. In the present study, we tried to elucidate the protective effect of TPP, which is known to have antioxidant properties, on IND-induced small intestinal damage by focusing on inflammation and oxidative stress.

The mechanism underlying the damage to membrane lipids of the small intestine during absorption of NSAIDs is the inhibition of lipid peroxidation and oxidative phosphorylation by TJP dysfunction. Furthermore, IND inhibits the synthesis of PGE, resulting in a lack of mucosal healing. For this reason, IND has been reported to be the NSAID that increases intestinal permeability the most. Increased permeability triggers damage to the small intestinal epithelium. Contact of luminal contents with damaged epithelium increases ROS. As a result of the sensitivity of the mucosal surface to ROS, this series of events triggering each other in the small intestine leads to disruption of the function (Handa et al. 2014; Tai and McAlindon 2018a). Previous studies have shown that antioxidant replacement in IND-induced small bowel injury reduces IND-related gastro-duodenal and small bowel damages (Sivalingam et al. 2007; Turkyilmaz et al. 2019; Wada et al. 2022; Sánchez-Trigueros et al. 2024). In the present study, the highest MDA and TOS levels in the IND group revealed the oxidative stress caused by IND in the small intestine. Pretreatment with TPP resulted in no decrease in MDA and TOS levels. GSH and antioxidant enzymes SOD and GPX levels were the lowest in the IND group and the highest in the TPP-treated groups, suggesting that TPP may have the potential to contribute to intracellular antioxidant levels. However, the TAS levels of the tissue were not significantly higher in the TPP-treated IND-injured group compared to the control and patient groups. These conditions suggested that it might be due to the short-term observation of the model and single-dose TPP administration. Because TPP supported to increase the antioxidant level and decreased the oxidative stress in terms of group averages. TPP has been found to prevent oxidative stress by preventing the decrease in antioxidant enzyme (SOD, GPX) levels in isoniazid and rifampicin-induced hepatic damage (Yeter et al. 2024). These findings supported each other with the results of the present study. Although there are no studies on the effect of TPP on IND-induced oxidative stress, some studies have revealed the effects of different drugs on oxidative stress in side effects or disease models. Again in different studies, it was reported that cisplatin, methotrexate, acetaminophen, and propofol showed antioxidant effects by reducing neutrophil infiltration, lipid peroxidation, DNA damage, and regulated liver function tests in hepatoxicity (Demiryilmaz et al. 2012; Turan et al. 2013b; Uysal et al. 2016; Delen et al. 2022). In addition, in a study examining the toxic effect of cisplatin in TPP testicular tissue, sperm motility, concentration, testosterone hormone levels, and enzymatic antioxidant levels were measured and reported to have antioxidant effects (Mnati et al. 2020).

Studies have shown that TPP has anti-inflammatory effects in reproductive system, heart, brain, and liver tissues (Turan et al. 2013a; Mahdavifard and Nakhjavani 2020; Delen et al. 2022). In an experimental study using a type 2 diabetes model, it was reported that thiamine (vitamin B1) showed an anti-inflammatory effect by direct administration (Mahdavifard and Nakhjavani 2020). However, in several studies examining the biological effects of thiamine and TPP, it was reported that thiamine did not yield significant results (Turan et al. 2013b). Upregulation of TNF-α and IL- 6 proinflammatory cytokines is involved in NSAID-induced gastrointestinal damage (Fukumoto et al. 2011; Abd-Ellatif et al. 2025; Nurliyani et al. 2025). Previous studies have reported increased expression of TNF-α, TLR4, and IL- 6 after IND small intestinal injury (Watanabe et al. 2008; Lin et al. 2022). Positive effects on the inflammatory status of sepsis rats fed TPP-enriched pellet feed have been previously reported (de Andrade et al. 2014). However, in the present study, we evaluated the inflammation status in the small intestinal tissue of IND-induced small intestine with TPP, which has not been investigated before. The role of inflammation in IND enteropathy was emphasized in small intestinal tissues in which TNF-α and IL- 6 levels were significantly higher than in the control group; however, the level of proinflammatory cytokines in the IND + TPP group pretreated with TPP before damage occurred was approximately the same as in the IND group, and no significant decrease was observed. Similar to our study, in a study examining the regulation of sepsis status with single-dose TPP administration, the anti-inflammatory effect of TPP was evaluated by looking at myeloperoxidase enzyme and a positive effect was reported through neutrophil infiltration (Giustina et al. 2019). However, this evaluation of TPP showing an anti-inflammatory effect via MPO with a single-dose administration is not compatible with our results in the present study. In fact, it was reported that TNF-α deficiency decreased IND-induced damage, and it was reported that TNF-α is an important cytokine in the evaluation of inflammation in IND damage (Scheiman 2016). The fact that the increase in TNF-α and IL- 6 levels, which are considered as the gold standard in the assessment of inflammation status, was reduced by TPP single-dose administration indicates that more detailed studies are needed.

In NSAID-induced small intestinal injury, it is known that disruption of the structures in the intestinal tissue leads to impaired function. When the cellular effects of the damage are examined, it is known that disruption of the villus structure, erosive findings, congestion, macrophage infiltration, and various cell death types are observed (Kunikata et al. 2002; Turkyilmaz et al. 2019; Cervantes-García et al. 2020; Han et al. 2020). In this study, the effects of pretreatment with TPP on indomethacin-induced small intestinal injury were investigated for the first time. In accordance with the biochemical parameters, microscopic examination revealed severe inflammation (Table 1). TPP pretreatment was found to reduce severe inflammation in the scoring obtained in the examination of inflammation. In addition, in the present study, IND-induced disruptions in the villus architecture of the small intestine were found to be quite severe, but pretreatment with a thesis dose of TPP failed to preserve tissue integrity (Fig. 5). In a study in which preventive treatment with Olea europaea was applied in IND-induced small intestine injury, it was reported that similar to the results of our study in crypt-to-villus ratio and villus architecture changes, the deterioration in the morphologic structure of the tissue was not prevented by preventive treatment (Mahdavi et al. 2020). However, in studies conducted by Sánchez-Trigueros et al. focusing on different topics, it has been reported that docosaenoic acid pretreatment has an important protective and therapeutic role in acute small intestinal injury (Sánchez-Trigueros et al. 2021, 2024).

The important limitation of this study is the paucity of studies examining IND-induced small bowel injury and the lack of a study focusing on TPP-induced intestinal enteropathy. However, these shortcomings make our study unique. In the present study, the increase in antioxidant status with TPP pretreatment in IND-induced small intestinal injury and the mild appearance of damage on microscopic examination were indicative of its positive effects. In addition, inflammation in the tissue was revealed by increased proinflammatory cytokine levels and histopathologic analysis. However, the fact that the damages were milder in the TPP pretreatment group in microscopic analyses shows that there are still researches that need to be explained here. Considering the previous studies, we thought that the dose of indomethacin may be too high in the model application, which may cause us not to see the full effect of TPP. Because no study was found for small intestinal damage of IND except the study of Turkyilmaz et al. (Turkyilmaz et al. 2019) at this dose. In previous studies, subcutaneous administration of IND as a model or by creating a chronic model (such as giving a dose of 6 mg/kg on certain days) was investigated (Fukumoto et al. 2011; Cervantes-García et al. 2020). It should be noted that the oral route of IND use in the clinic and the development of enteropathy in the small intestine with subcutaneous IND administration is a pharmacological limitation or incompatibility. Indeed, our study sheds light on the researchers who aim to examine the effects by creating an acute model by adjusting the doses and durations in the experimental model.

In conclusion, the antioxidant and anti-inflammatory effects of TPP supporting the information in the literature were also shown in this study. However, we think that it would be more enlightening to apply a longer-term experimental procedure and to investigate the pharmacological effects of TPP in detail in order to reveal the events occurring in cellular damages with molecular pathways.

## Data Availability

All source data for this work (or generated in this study) are available upon reasonable request.
